# Trends in medication adherence in HIV patients in the US, 2001 to 2012: an observational cohort study

**DOI:** 10.1002/jia2.25382

**Published:** 2019-08-23

**Authors:** Bora Youn, Theresa I Shireman, Yoojin Lee, Omar Galárraga, Ira B Wilson

**Affiliations:** ^1^ Department of Health Services, Policy & Practice Brown University School of Public Health Providence RI USA

**Keywords:** adherence, anti‐retroviral agents, trend, United States, Medicaid, HIV

## Abstract

**Introduction:**

Adherence to antiretroviral therapy (ART) is essential to reduce HIV‐related morbidity and mortality as well as the risk of virological failure and HIV transmission. We determined the trends in ART adherence during the periods of therapeutic advances, wider use of ART and greater attention to ART adherence. To understand the general trends in medication adherence, we compared ART adherence with medications for other common chronic conditions.

**Methods:**

A retrospective cohort study using Medicaid claims between 2001 and 2012 from 14 US states with the highest HIV prevalence. Medicaid is the largest source of care for HIV patients in the US. We identified Medicaid beneficiaries with HIV who initiated ART between 2001 and 2010 (n=23,343). Comparison groups included (1) HIV‐ persons who initiated a statin, angiotensin‐converting enzyme inhibitor or angiotensin receptor blocker (ACEI/ARB), or metformin and (2) HIV+ persons who initiated these control medications while on and not on ART. We estimated adjusted odds of >90% medication implementation during the two years following initiation.

**Results:**

The proportion of HIV+ persons with >90% ART implementation increased from 33.5% in those who initiated in 2001 to 46.4% in 2005 and 52.4% in 2010. ART initiators in 2007 to 2010 had 53% increased odds of >90% implementation compared to those in 2001 to 2003 (adjusted OR 1.53, 99% CI: 1.34 to 1.75). Older age, male, White race, newer ART regimens and absence of substance use indicators were also associated with increased odds of >90% ART implementation. No or minimal improvements were found in the implementation of control medications in HIV‐ persons. For HIV‐ persons, the adjusted ORs comparing 2007–2010 to 2001–2003 were 1.06, 1.01 and 1.19 for statins, ACEI/ARB, metformin respectively. HIV+ persons who were on ART had, on average, 15.0 (SD: 4.2) and 16.1 (SD: 3.4) percentage points higher >90% implementation rates of concurrent statins, ACEI/ARB or metformin compared to HIV‐ persons and HIV+ persons who were not on ART respectively.

**Conclusions:**

Adherence to ART substantially improved between 2001 and 2012. Nevertheless, the absolute rates of >90% implementation were low for all groups examined. Substantial disparities by age, sex and race were present, drawing attention to the need to continue to enhance medication adherence. Further studies are required to examine whether these trends and disparities persist in the most recent period.

## 1 Introduction

The management of HIV infection has changed substantially in the past three decades with the introduction of novel antiretroviral therapy (ART) with improved tolerability and convenience. In the US, all HIV‐positive (HIV+) adults have been recommended to initiate ART since 2012 1, 2. For patients who can maintain adequate levels of adherence to these effective treatments, HIV infection can be transformed into a manageable chronic condition 3, 4, 5.

Many patients with asymptomatic chronic conditions, such as early stage HIV infection, have difficulty adhering to their recommended medication regimens 6. Suboptimal adherence to ART is particularly problematic because of increased HIV‐related morbidity and mortality, as well as the risk of drug resistance and HIV transmission to uninfected people 5, 7. Accordingly, numerous interventions have been employed to improve medication taking 8. In the US, almost all HIV care providers reported discussing ART adherence at every visit, and more than half of them referred non‐adherent patients for adherence support services 9.

Medication adherence consists of three phases: initiation, persistence and implementation 10. Persistence and implementation have often been conflated in prior studies although they represent related but different patient behaviours 7. Persistence refers to continuous treatment with a prescribed medication, whereas implementation refers to the extent to which a patient follows a prescribed dosing regimen while remaining on treatment 10. In a previous study, we reported improved persistence with ART among HIV+ persons with Medicaid between 2001 and 2010 11. Median time to ART non‐persistence increased from 23.9 months in 2001–2003 to 35.4 months in 2004–2006 but was not reached for those initiating ART in 2007–2010 due to lack of follow‐up after 2010.

In this study, we determined trends in ART implementation in a large population‐based cohort of US Medicaid beneficiaries with HIV. We included data from 2001 to 2012 to understand the trend in ART implementation during the periods of newer ART regimens, wider use of ART and greater attention to adherence 4, 12. To understand the contextual factors that may have influenced secular trends in medication implementation, we compared ART implementation among HIV+ persons with the implementation of control medications (statins, angiotensin‐converting enzyme inhibitor (ACEIs) or angiotensin receptor blocker (ARBs) or metformin) among HIV+ and HIV‐negative (HIV‐) persons.

## 2 Methods

### 2.1 Data sources

We used the Medicaid Analytic eXtract (MAX) files between 2001 and 2012 from 14 US states, which account for 75% of the HIV prevalence in the US. These states are New York, California, Florida, Texas, Maryland, New Jersey, Pennsylvania, Illinois, Georgia, North Carolina, Virginia, Louisiana, Ohio and Massachusetts 13. Medicaid is the largest public health insurance programme for low‐income individuals in the US and the largest source of care for HIV patients 14.

### 2.2 Study population

We identified three groups of patients: HIV+ persons who initiated ART (Group A); HIV‐ persons who initiated a statin, ACEI/ARB or metformin (Group B); and HIV+ persons who initiated statin, ACEI/ARB or metformin (Group C) between 2001 and 2010 in the Medicaid fee‐for‐service system. The HIV status of each patient was determined as previously described 11. HIV+ persons who initiated statin, ACEI/ARB or metformin were further classified as (1) starting the medication while on ART (Group C1) or as (2) using the medication while not on ART (Group C2).

To ensure complete assessment of medication initiation and outcome, we included beneficiaries with Medicaid fee‐for‐service coverage from the six months prior to their first fill (i.e. wash‐out period) to two years after initiation (i.e. follow‐up period). We excluded beneficiaries in Medicaid managed care plans, those dually eligible for Medicare, and those who held multiple state eligibility status because we may not have the full prescription claims of these persons. Beneficiaries with less than one month of use of respective medications were also excluded. For HIV+ beneficiaries using ART (Group A), those with incomplete ART regimen usage, which was defined as less than three different antiretroviral ingredients, were excluded. For HIV+ beneficiaries who were also using control medications (Group C), we included only those who met criteria for having HIV prior to their statin, ACEI/ARB or metformin first fill date.

### 2.3 Adherence assessment

We used the definition and methods recommended in the EMERGE guidelines to assess medication adherence during the two years following initiation 15. To generate the implementation rate for each medication, we used days supplied and fill dates within each therapeutic class at the patient level. The denominator was the number of days from the first prescription date until (1) the last prescription date for those who became non‐persistent within two years or (2) two‐years after initiation (730 days) for those who remained persistent. The numerator was the sum of number of days of medication supplied. Figure S1 illustrates the detailed methods of implementation measurement.

Implementation rates were dichotomized using a 90% cutoff (two‐year fully implemented yes/no), with sensitivity analyses as described below. Recent studies have shown that lower levels of adherence may be sufficient for newer ART regimens, as compared to 95% required for older ART regimens 16.

To allow for switching (e.g. switching ART ingredients, switching from ACEI to ARB), patients continued to accumulate days of medication use as long as they continued to receive an agent from the same therapeutic class.

### 2.4 Study variables

To characterize time trends, the primary exposure of interest was the calendar year of medication initiation. Initiation years were classified into three groups (2001 to 2003, 2004 to 2006, 2007 to 2010). Patient demographics included age group, sex, race/ethnicity and state. We also developed variables for substance use, including indicators for alcohol, drug and tobacco use based on International Classification of Diseases‐9 codes, Current Procedural Terminology codes and Diagnosis Related Group codes 17, 18, 19, 20, 21. We considered substance use as time‐invariant.

For ART users (Group A), we controlled for the index regimen characteristics including (1) nucleoside reverse transcriptase inhibitor (NRTI) backbone, (2) third‐drug composition and (3) ART daily pill burden. The NRTI backbones were classified into four categories: (1) didanosine (DDI) or stavudine (D4T), (2) zidovudine (ZDV), (3) abacavir (ABC) or tenofovir (TDF) and (4) others 22. We classified the three‐drug composition in the regimen as: (1) triple NRTI‐based regimens, (2) non‐nucleoside reverse transcriptase inhibitor (NNRTI)‐based regimens, (3) protease inhibitor (PI)‐based regimens, (4) boosted PI‐based regimens, (5) integrase inhibitor‐based regimens and (6) multiple classes. For ACEI/ARB users in Group B and C, we controlled for the type of index class used.

### 2.5 Statistical analysis

Our analytic approach had two parts. First, to determine whether general medication adherence secular trends contributed to ART implementation trends, we compared ART implementation with implementation of control medications (statins, ACEI/ARBs and metformin). Second, to determine whether engagement with ART therapy is associated with the implementation of non‐ART medications, we compared the implementation of control medications in HIV+ persons on and not on ART.

We computed descriptive statistics of HIV+ persons initiating ART by calendar year of initiation. For persons with statins, ACEI/ARBs or metformin, we assessed the differences in baseline characteristics by different medication classes and HIV status. For unadjusted analyses, we assessed whether the proportion of persons with >90% implementation changed over time. For control medications, we obtained the differences in proportions of HIV‐ and HIV+ persons with >90% implementation in each year and calculated the average difference across years. For adjusted analysis, we implemented multivariable logistic models for each medication and included patient and regimen characteristics, and treatment initiation year. For statins, ACEI/ARBs and metformin, we also included indicators for HIV status and interaction between HIV status and treatment initiation year. We assessed statistical significance using two‐sided tests at the 99% confidence level. We performed the analyses using SAS version 9.4 (SAS Institute, Cary, NC, USA). The Brown University Institutional Review Board approved this study.

### 2.6 Sensitivity analysis

We performed sensitivity analyses to ensure robustness of our findings. First, we used alternative cut‐offs (95% and 80%) to dichotomize persons who fully implemented each treatment. Second, we calculated an alternative adherence measure, the proportion of days covered (PDC). The denominator of PDC analysis was set to 730 days regardless of persistence status during the two‐year follow‐up 23. Third, we examined 1‐year implementation rates and implementation rates during the whole persistent episode (e.g. followed persons until the last prescription date, end of the study, death or loss of Medicaid coverage, whichever came first). Fourth, we obtained adjusted predicted rates of >90% implementation using marginal standardization 24. Fifth, we added patient comorbid conditions to the ART model 25. Sixth, we modified the substance use definition to those who had claims prior to their last medication fill date or the end of two‐year follow‐up period. Finally, we did not exclude HIV+ persons with incomplete ART regimen usage.

## 3 Results

### 3.1 Study patients

We identified 23,343 persons with HIV who initiated ART between 2001 and 2010 and met study eligibility criteria (Group A, Table 1, Figure S2). The majority were aged 35 to 54 years (67.0%), Black race (55.8%) and living in New York (36.7%), California (14.7%) or Florida (10.3%). The types of ART regimen used substantially changed over time with increasing use of TDF/ABC NRTI backbone, regimens with lower pill burden and regimens that included integrase inhibitors.

**Table 1:** Baseline characteristics of HIV‐positive persons with antiretroviral therapy by treatment initiation year

	All years (n = 23,343)	2001 to 2003, (n = 10,972)	2004 to 2006, (n = 6769)	2007 to 2010, (n = 5602)
Age, years (%)				
<25	8.6	8.4	8.8	8.7
25 to 34	15.9	16.6	14.9	15.5
35 to 44	37.0	40.8	36.5	30.2
45 to 54	30.0	27.9	30.5	33.5
55+	8.6	6.4	9.3	12.2
Sex (% male)	53.1	52.7	53.4	53.6
Race/ethnicity (%)				
Black	55.8	54.1	54.9	60.3
White	17.8	17.4	18.7	17.6
Hispanic	19.1	21.4	19.1	14.4
Asian/Pacific Islander/Native American	1.0	0.9	1.0	1.2
Multiracial/unknown	6.3	6.2	6.4	6.5
State (%)				
California	14.7	13.0	15.9	16.6
Florida	10.3	10.2	10.4	10.3
Georgia	6.0	4.6	5.1	9.9
Illinois	8.2	6.2	7.9	12.6
Louisiana	4.4	3.0	3.9	7.9
Massachusetts	4.8	4.0	6.2	4.6
Maryland	0.7	0.6	0.6	1.1
North Carolina	5.8	3.8	6.4	9.1
New Jersey	3.0	3.3	4.1	1.2
New York	36.7	45.7	34.2	21.9
Ohio	1.2	1.6	1.2	0.3
Pennsylvania	0.7	0.4	0.5	1.4
Texas	3.2	3.3	3.4	2.9
Virginia	0.4	0.4	0.3	0.3
NRTI backbone (%)				
TDF/ABC	38.2	9.8	48.2	81.8
ZDV	41.3	56.4	39.1	14.6
DDI/D4T	18.9	32.2	11.2	2.2
Others	1.6	1.7	1.5	1.4
Regimen type (%)				
Boosted PI based	29.5	20.3	35.7	40.0
PI based	17.4	22.1	16.7	9.3
Integrase Inhibitor based	0.5	0.0	0.0	2.2
NNRTI based	38.1	35.1	38.6	43.1
NRTI based	10.4	17.7	5.4	2.0
Multiple/others	4.1	4.7	3.6	3.5
ART pill burden (%)				
1	9.2	0	3.0	34.5
2 to 3	29.2	23.6	36.1	31.8
4 to 5	24.5	22.9	30.6	20.2
6 to 9	21.7	25.0	24.3	12.0
10+	15.5	28.5	6.0	1.6
Substance use (% yes)				
Alcohol use	42.2	44.2	42.2	38.5
Drug use	54.3	56.9	54.1	49.7
Tobacco use	38.4	37.6	39.1	38.9

Missing values accounted for 0.08% of sex and 0.06% of pill burden. Percentage may not sum to 100 because of rounding. ABC, abacavir; ART, antiretroviral therapy; D4T, stavudine; DDI, didanosine; NNRTI, non‐nucleoside reverse transcriptase inhibitor; NRTI, nucleoside reverse transcriptase inhibitor; PI, protease inhibitor; TDF, tenofovir; ZDV, zidovudine.

The characteristics of those who initiated statin, ACEI/ARB or metformin are described for HIV‐ (Group B, Table 2) and HIV+ persons (Group C, Table S1). Cohort selection diagrams are described in Figures S3, S4, S5.

**Table 2:** Baseline characteristics of HIV‐negative persons who initiated statins, angiotensin‐converting enzyme inhibitors/angiotensin receptor blockers or metformin

	Statins, (n = 359,245)	ACEI/ARB, (n = 371,204)	Metformin, (n = 180,538)
Treatment initiation year (%)			
2001 to 2003	32.1	34.0	29.2
2004 to 2006	33.7	31.3	30.8
2007 to 2010	34.2	34.8	40.0
Age, years (%)			
<25	2.7	5.7	11.9
25 to 34	7.1	9.2	11.9
35 to 44	19.0	19.7	20.2
45 to 54	35.7	33.5	29.7
55+	35.5	31.9	26.3
Sex (% male)	36.1	38.0	31.9
Race/ethnicity (%)			
Asian/Pacific Islander/Native American	5.3	4.2	3.9
Black	23.8	31.7	28.7
Hispanic	16.1	15.3	21.5
Multiracial/Unknown	8.7	8.1	7.5
White	46.0	40.9	38.3
State (%)			
California	24.5	23.7	22.9
Florida	7.3	6.5	6.3
Georgia	6.3	7.0	6.5
Illinois	13.6	14.4	15.0
Louisiana	5.5	7.1	5.9
Massachusetts	5.6	4.6	4.8
Maryland	0.2	0.2	0.4
North Carolina	7.9	8.7	9.0
New Jersey	1.2	1.2	1.1
New York	11.3	9.9	10.5
Ohio	5.2	4.9	4.8
Pennsylvania	3.0	2.6	2.7
Texas	7.0	7.9	8.7
Virginia	1.5	1.4	1.4
Index regimen type (%)			
ACEI	‐	75.6	‐
ARB	‐	24.1	‐
ACEI + ARB	‐	0.3	‐
Substance use (% yes)			
Alcohol use	21.5	23.8	20.8
Drug use	22.4	24.5	22.4
Tobacco use	25.4	25.0	21.7

Missing values in sex accounted for 0.03%, 0.04% and 0.06% of statin, ACEI/ARB and metformin initiators respectively. Percentage may not sum to 100 because of rounding. ACEI, angiotensin‐converting enzyme inhibitor; ARB, angiotensin receptor blocker.

### 3.2 Time trends in implementation of ART and control medications

**Figure 1:**
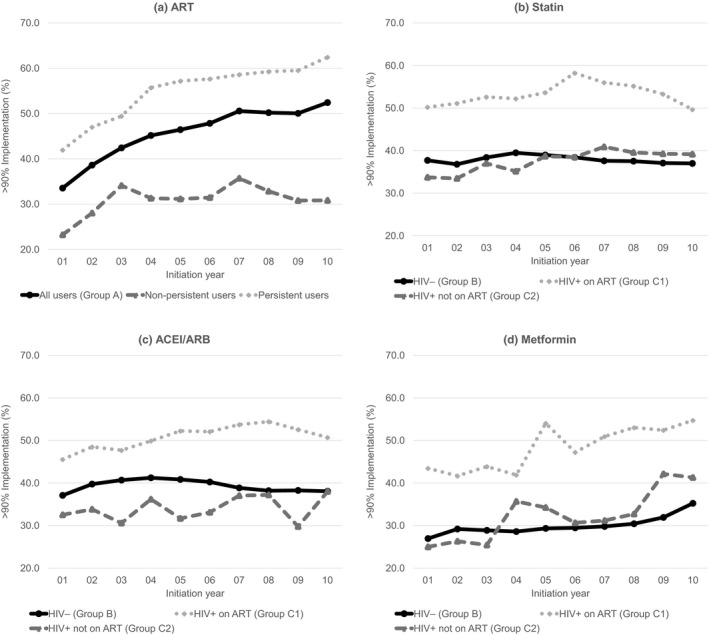
Unadjusted trends of >90% implementation during the two years following initiation For ART analyses: All users include 23,343 HIV+ persons who initiated ART between 2001 and 2010. Persistent users include 13,749 HIV+ persons who remained persistent with ART during the two years following initiation. Non‐persistent users include 9594 HIV+ persons who became non‐persistent. ACEI, angiotensin‐converting enzyme inhibitor; ARB, angiotensin receptor blocker; ART, antiretroviral therapy.

Two‐year implementation of ART substantially improved over time (Figure 1a). The percentage of HIV+ persons with >90% ART implementation increased from 33.5% in persons who initiated in 2001 to 46.4% in 2005 and 52.4% in 2010, an improvement of 18.9 percentage points. Approximately half of those who initiated ART (58.9%) remained persistent during the two years following initiation. Trends towards improved implementation existed in both persistent and non‐persistent ART users (Figure 1a).

Trends in implementation for statins, ACEI/ARBs and metformin are shown in Figure 1, Panels b, c and d. The implementation trends for HIV‐ persons are similar to the trends for HIV+ persons who are not on ART. In contrast, the trend for HIV+ persons who start the medication while on ART are, on average, 16.1 percentage points higher than HIV+ persons without ART (standard deviation (SD): 3.4) and 15.0 percentage points higher than HIV‐ persons (SD: 4.2) each year.

### 3.3 Variables associated with >90% implementation

Table 3 shows the results of a multivariable model predicting >90% ART implementation. ART implementation clearly increased over the time period examined. Those who initiated ART between 2007 and 2010 had 53% increased odds of >90% implementation compared to those initiated between 2001 and 2003 (odds ratio (OR) 1.53, 99% confidence interval (CI): 1.34 to 1.75). Factors that were associated with higher odds of >90% ART implementation include older age, male, White race, living in New York or California, newer ART regimens, lower ART pill burden and absence of substance use indicators.

**Table 3:** Multivariable predictors of the odds of >90% implementation of antiretroviral therapy

	Adjusted OR (99% CI)
Treatment initiation year (ref=2001 to 2003)	
2004 to 2006	1.29 (1.17, 1.43)
2007 to 2010	1.53 (1.34, 1.75)
Age, years (ref = 55+)	
<25	0.77 (0.65, 0.92)
25 to 34	0.59 (0.51, 0.69)
35 to 44	0.69 (0.61, 0.79)
45 to 54	0.82 (0.72, 0.94)
Sex (ref = Female)	
Male	1.10 (1.02, 1.18)
Race/Ethnicity (ref = White)	
Black	0.73 (0.66, 0.81)
Hispanic	0.92 (0.82, 1.04)
Asian/Pacific Islander/Native American	1.10 (0.77, 1.57)
Multi/unknown	0.84 (0.71, 0.99)
State (ref = New York)	
California	1.04 (0.93, 1.17)
Florida	0.60 (0.53, 0.68)
Georgia	0.36 (0.30, 0.43)
Illinois	0.81 (0.71, 0.93)
Louisiana	0.48 (0.40, 0.58)
Massachusetts	0.57 (0.48, 0.68)
Maryland	0.42 (0.27, 0.66)
North Carolina	0.57 (0.49, 0.67)
New Jersey	0.79 (0.64, 0.98)
Ohio	0.67 (0.48, 0.94)
Pennsylvania	0.81 (0.53, 1.26)
Texas	0.25 (0.20, 0.32)
Virginia	0.41 (0.21, 0.80)
NRTI backbone (ref=TDF/ABC)	
ZDV	0.92 (0.83, 1.02)
DDI/D4T	0.89 (0.78, 1.02)
Others	0.64 (0.46, 0.88)
Regimen type (ref=PI based)	
Boosted PI based	0.98 (0.87, 1.11)
Integrase inhibitor based	1.83 (1.08, 3.08)
NNRTI based	1.16 (1.01, 1.33)
NRTI based	0.88 (0.73, 1.06)
Multiple class	1.21 (0.99, 1.49)
ART pill burden (ref=10+)	
1	1.27 (0.98, 1.65)
2 to 3	1.32 (1.10, 1.57)
4 to 5	1.15 (0.99, 1.34)
6 to 9	1.11 (0.97, 1.26)
Alcohol use (ref=no)	0.85 (0.78, 0.94)
Drug use (ref=no)	0.90 (0.82, 0.98)
Tobacco use (ref=no)	0.90 (0.83, 0.98)

Results from a multivariable logistic regression model with all variables listed in the table. Variable selection was based on predictors of adherence in prior literature. ABC, abacavir; ART, antiretroviral therapy; CI, confidence interval; D4T, stavudine; DDI, didanosine; NNRTI, nonnucleoside reverse transcriptase inhibitor; NRTI, nucleoside reverse transcriptase inhibitors; OR, odds ratio; PI, protease inhibitor; Ref, reference category; TDF, tenofovir; ZDV, zidovudine.

**Figure 2:**
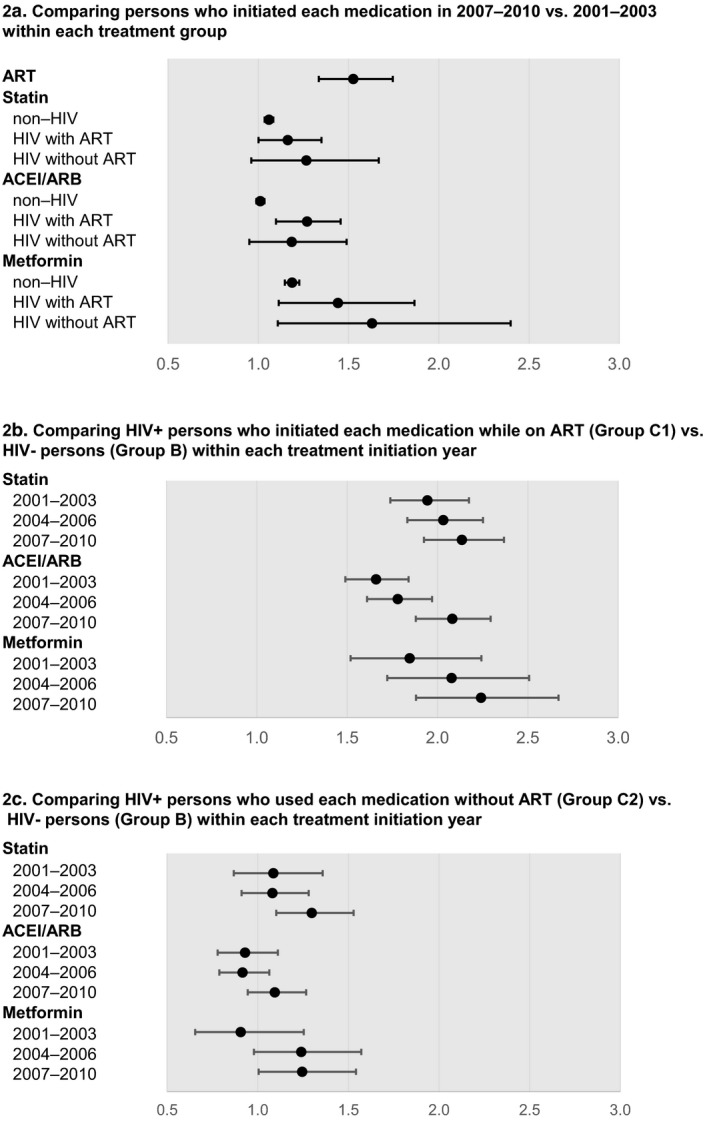
Adjusted odds of >90% implementation of ART, statin, ACEI/ARB and metformin (**a**) Comparing persons who initiated each medication in 2007 to 2010 versus 2001 to 2003 within each treatment group. (**b**) Comparing HIV+ persons who initiated each medication while on ART (Group C1) versus HIV‐ persons (Group B) within each treatment initiation year. (**c**) Comparing HIV+ persons who used each medication without ART (Group C2) versus HIV‐ persons (Group B) within each treatment initiation year. Odds ratios from four logistic regression models developed for each medication. All models included age, sex, race, state, substance abuse and treatment initiation year. ART model additionally controlled for ART regimen characteristics. Models for statin, ACEI/ARB and metformin additionally included HIV group status and their interaction with treatment year. ACEI/ARB model additionally included ACEI/ARB regimen type. ART, antiretroviral therapy; ACEI, angiotensin‐converting enzyme inhibitor; ARB, angiotensin receptor blocker.

Figure 2a describes the adjusted odds of control medication implementation between those in 2007 to 2010 and 2001 to 2003. For HIV‐ persons (Group B), the ORs comparing 2007–2010 to 2001–2003 were 1.06 (CI: 1.04–1.08) for statins, 1.01 (CI: 0.99 to 1.03) for ACEI/ARBs and 1.19 (CI: 1.15 to 1.23) for metformin. The odds of >90% implementation increased over time among HIV+ persons with or without ART.

Figure 2b and 2c compare the adjusted odds of >90% implementation between groups C1 and B (Figure 2b) and C2 and B (Figure 2c) within each year group. Among 2007 to 2010 initiators, the odds of >90% implementation comparing HIV+ persons on ART (Group C1) versus HIV‐ persons (Group B) were 2.14 for statins (CI: 1.93 to 2.37), 2.08 for ACEI/ARBs (CI: 1.89 to 2.29) and 2.24 for metformin (CI: 1.88 to 2.67) respectively (Figure 2b). In contrast, among 2007 to 2010 initiators, the odds of >90% implementation comparing HIV+ persons not on ART (Group C2) versus HIV‐ persons (Group B) were similar, except for those who initiated statin in 2007 to 2010 (OR 1.30, CI: 1.10 to 1.53) (Figure 2c).

### 3.4 Sensitivity analyses

First, implementation trends and predictors were similar when 95% and 80% cutoffs were used (Table S2). Second, when we used PDC to measure adherence during two years following initiation, the proportion of persons with PDC higher than 90% increased from 20.0% in 2001 to 29.7% in 2005 and 39.7% in 2010 (Table S2). The adjusted odds of >90% PDC were 25% higher comparing 2007 to 2010 initiators to 2001 to 2003 initiators (OR 1.25, CI: 1.08 to 1.45) (Table S3). Third, our results were robust when we used different follow‐up time for the denominator, 1‐year implementation rate and implementation rate during the whole persistent episode (Table S2). Fourth, the adjusted predicted rate of >90% ART implementation increased from 34.8% in 2001 to 45.8% in 2005 and 54.4% in 2010 initiators (Table S2). Fifth, adding comorbid conditions to the ART model did not change the results (results available upon request). Sixth, the results of the adjusted models remain similar when we used different observation periods to define substance use status (results available upon request). Finally, the results were similar when we included HIV+ persons with incomplete ART regimen usage (results available upon request).

## 4 Discussion

This research has three main findings. First, ART implementation substantially improved between 2001 and 2012, even after accounting for changes in patient and regimen characteristics. No or minimal improvements were found in the implementation of control medications in HIV‐ persons, providing strong evidence that the changes in ART cannot be solely explained by general secular trends. Second, the implementation of control medications in HIV+ persons not on ART were similar to HIV‐ persons, whereas >90% implementation in those on ART was, on average, 16.1 percentage points higher than those without ART. Engagement with ART care seems to be associated with improved implementation of non‐HIV related medications. Third, the absolute rates of >90% implementation were low for all groups and medications examined – especially for blacks, younger age groups and those in certain states.

The findings presented in this study extend our previous work on ART persistence between 2001 and 2010 in four main ways 11. First, and most importantly, the papers examine fundamentally different adherence concepts. Persistence is the time to treatment discontinuation, whereas implementation is the degree to which a medication is taken as directed during persistent periods 7, 10. This distinction has been emphasized in a recent paper proposing guidelines for papers on medication adherence 15. Although the odds and hazard ratio estimates from different studies cannot be directly compared, the magnitude of improvement observed for implementation analyses was greater than that for persistence analyses. Second, in this study we were able to use two more years of data, 2011 and 2012, which furthers our understanding of implementation rates during the periods of wider use of ART 1. Third, in the analysis we accounted for substance use status, which was not available in our previous study because of the federal regulations that have since changed 26. Fourth, the predictors of these different adherence outcomes were also slightly different. For example, integrase‐inhibitor‐based regimens were significantly associated with >90% implementation but not with persistence.

There are limited nationally representative data that examine time trends in ART adherence in the US 27. In the Veterans Aging Cohort Study virtual cohort (98% men), ART adherence increased by 13% every two years on average between 2001 and 2010 28. In the Women's Interagency HIV Study (100% women), self‐reported 95% adherence increased from 78% in 2006 to 85% in 2013 29. In the Centers for AIDS Research Network of Integrated Clinical Systems (CNICS) cohort, which used a self‐report measure, mean adherence did not increase between 2010 and 2015 4. The differences in findings may be attributable to the differences in adherence assessment method, study period and patient population. The CNICS cohort includes patients receiving care at eight HIV clinics associated with academic medical centres 30, whereas patients with Medicaid comprise low‐income patients receiving care in routine practice. Because we examined Medicaid beneficiaries in the 14 states with the highest HIV prevalence over a 12 year time period, we believe that our findings are the most comprehensive and generalizable to date.

Our use of control medications in HIV‐ persons allows us to assert that the observed 18.9 percentage point improvement in >90% ART implementation was not the result of secular trends. One potential explanation of this improvement is that there were changes in the sociodemographic or clinical characteristics of the population, or that newer, more effective and less toxic ART regimens came into use. However, our adjusted analyses controlled for most of these factors.

Another factor is changes in ART initiation guidelines between 2001 and 2012. The US Department of Health and Human Services guideline recommended initiating ART for those with CD4 count <200 cells/mm^3^ in 2001 31, and subsequently increased the threshold to <350 cells/mm^3^ in 2007 32, <500 cells/mm^3^ in 2009 33 and to all HIV+ adults regardless of CD4 count in March 2012 1. The International Antiviral Society‐USA guideline also recommended initiating ART in all HIV+ adults in 2012 2. Although HIV+ persons in recent periods have likely initiated ART at a higher CD4 count on average than those in earlier periods 12, a recent systematic review found no consistent association between baseline CD4 count and ART adherence in routine clinical settings 34. When we separately examined patients who initiated ART in 2012 (Table S2, implementation rate during persistent episode), the estimate of ART implementation rate and its trend remained similar between those in 2012 and those in the immediately preceding years.

Another potential explanation for improved medication implementation among HIV+ persons is the presence of adherence support services at HIV care sites. Since 2001, federal HIV treatment guidelines have recommended monitoring ART adherence at every clinical visit 31. Accordingly, approximately half of the HIV care sites in the US provided programmes specifically designed to support patients’ adherence to ART between 2009 and 2011 35, and one in five HIV patients reported using adherence support services 27. The quality of routine adherence care in clinical sites significantly affect the medication‐taking behaviour of patients 36, and increased attention to medication adherence in HIV care sites may explain the improvements in medication implementation observed for HIV+ persons.

Our examination of HIV+ persons on and not on ART revealed that the >90% implementation of control medications was, on average, 16.1 percentage points higher for those using ART compared to those not using ART. For HIV+ persons using ART, engagement with both care and treatment for HIV may make it easier, both practically and behaviourally, to engage with medication treatments for other chronic conditions. However, we note that previous studies on the effect of multiple medication use on adherence were inconclusive 37, 38, 39. It is also possible that ART adherence support interventions have spillover effects on other chronic medications. Studies that attempt to understand the drivers of this improved implementation are needed. In contrast, our findings of no or minimal improvements of control medication implementation among HIV‐ persons are similar to the results of prior studies 40, 41, and are likely to reflect the challenges of improving medication taking behaviour 41.

Despite the improvements, the absolute rates of >90% implementation remain low, never exceeding 60% for any medication that we examined. Consistent with our prior research, Blacks, women, younger age groups, patients with substance use and those living in Georgia and Texas were more likely to have lower rates of >90% ART implementation, possibly due to limited resources, restricted access to care and high levels of HIV‐related stigma 27, 42, 43. Differences between states may also be attributable to differences in Medicaid programme generosity, including Medicaid eligibility criteria, out‐of‐pocket spending, access to providers and limits on the number of reimbursable prescriptions 44. For example, Texas and Georgia Medicaid programmes limited the number of prescriptions to three and five per month during our study period respectively 45.

Our analysis has several limitations. First, our data do not extend beyond 2012 because Medicaid data for the years after 2012 are not yet available for many states. Nevertheless, our findings from more recent periods may be generalizable to the current era of treating all HIV+ persons, given the changes in US‐based guidelines in 2012 and the availability of observational studies suggesting the benefit of early ART initiation since 2009 1, 46. Nevertheless, further studies are necessary to determine whether these trends of improved implementation persist after 2012. Second, we did not have information on CD4 counts and viral loads to examine the effect of improved ART implementation on other outcomes. Nevertheless, our findings are generally aligned with improved HIV care in the US in the past two decades, including early diagnosis, timely ART initiation, viral suppression and reduced number of new HIV diagnoses 12, 47, 48. Future studies can examine how these factors have influenced our findings, and their subsequent effects on improved clinical outcome and reduced HIV transmission. Third, patients who initiated ART at different time points may have differed in unmeasured characteristics. Fourth, as our results are based upon Medicaid fee‐for‐service enrollees, they may not be generalizable to other HIV+ population. We also excluded prevalent ART users to improve the internal validity of study findings 49. Finally, the presence of a medication claim does not necessarily indicate that the patient took the medication.

## 5 Conclusions

In conclusion, implementation of ART improved over the 12‐year period that we examined, although it is lower than desired for these highly effective medications. Substantial disparities by age, sex, race and state were present, even within this insured population who by definition has limited resources. Further studies are needed to examine whether these trends and disparities persist in the most recent period. Our results highlight the continued importance of interventions and policies to help patients take medications and optimize health outcomes, particularly among those with low levels of adherence. Although reasons for not taking medication are complex, clinicians and health systems can implement effective strategies in the context of routine clinical care 50. These strategies should take account of the fact that effective ART use requires a series of related but separate behaviours: timely initiation, long‐term persistence and high levels of effective implementation while persistent 10.

## Competing interests

All authors declare that they have no competing interests.

## Authors’ contributions

BY, TS and IW designed the study. BY and YL contributed to data analyses. BY and IW wrote the first draft of the manuscript. All authors contributed to interpretation of data, critically reviewed the manuscript and agreed on its final version.

## Supporting information


**Figure S1.** Illustration of implementation rate calculation methods.
**Figure S2.** Construction of the analytic sample of HIV+ persons with antiretroviral therapy.
**Figure S3.** Construction of the analytic sample of HIV− and HIV+ persons with statin.
**Figure S4.** Construction of the analytic sample of HIV− and HIV+ persons with ACEI/ARB.
**Figure S5.** Construction of the analytic sample of HIV− and HIV+ persons with metformin.
**Table S1.** Baseline characteristics of HIV+ persons who initiated statin, ACEI/ARB or metformin
**Table S2.** Trends in antiretroviral therapy adherence using different outcome measurements
**Table S3.** Adjusted odds of >90% proportion of days covered
