# Supplementary material for: Trends in medication adherence in HIV patients in the US, 2001 to 2012: an observational cohort study
Source: J Int AIDS Soc. 2019 Aug 23;22(8):e25382. doi: 10.1002/jia2.25382 (PMC6706701; doi:10.1002/jia2.25382)
Supplement: Supplementary file 1 — Figure S1. Illustration of implementation rate calculation methods. Figure S2. Construction of the analytic sample of HIV+ persons with antiretroviral therapy. Figure S3. Construction of the analytic sample of HIV− and HIV+ persons with statin. Figure S4. Construction of the analytic sample of HIV− and HIV+ persons with ACEI/ARB. Figure S5. Construction of the analytic sample of HIV− and HIV+ persons with metformin. Table S1. Baseline characteristics of HIV+ persons who initiated statin, ACEI/ARB or metformin Table S2. Trends in antiretroviral therapy adherence using different outcome measurements Table S3. Adjusted odds of >90% proportion of days covered [file JIA2-22-e25382-s001.docx]

[Appendix Figure 1] Illustration of implementation rate calculation methods


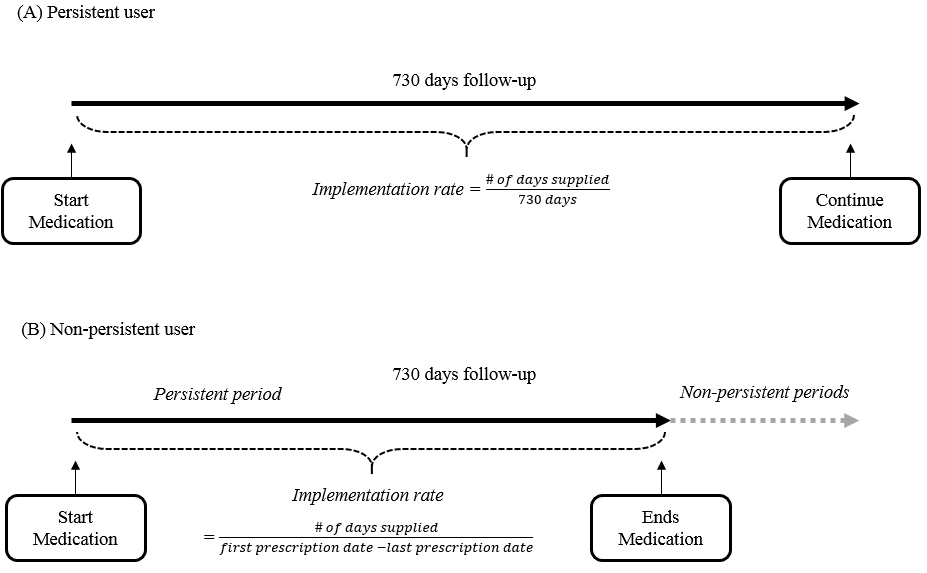


A person was considered to be persistent if they continuously filled respective medications for two-years without exceeding a 90-days permissible gap. A permissible gap is the maximum number of consecutive days that a patient can miss medications before being classified as non-persistent [1]. For persons who re-initiated treatment after a 90-days permissible gap during the follow-up period, we included only the first persistent episode in the analysis.

[Appendix Figure 2] Construction of the analytic sample of HIV+ persons with antiretroviral therapy


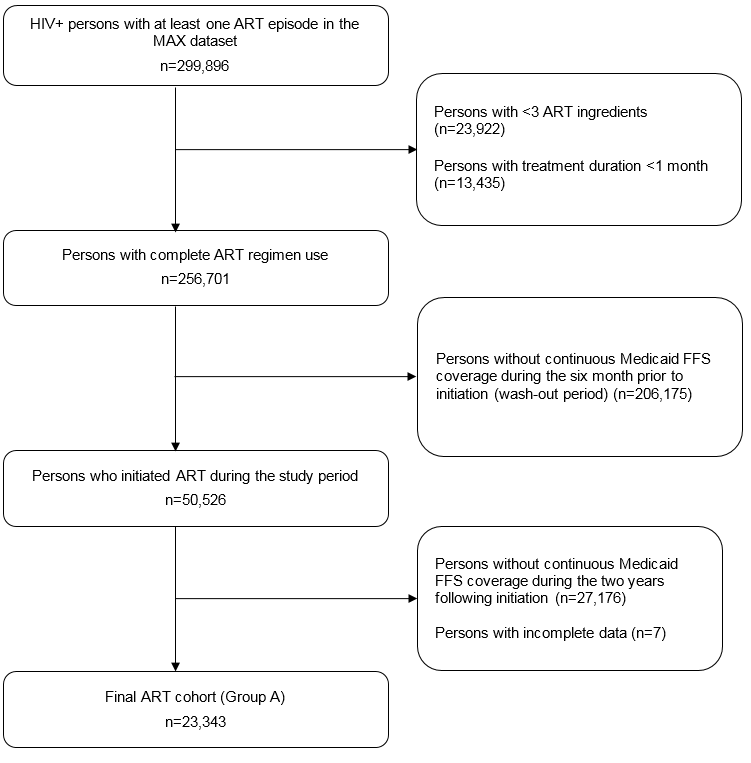


Abbreviations: ART, antiretroviral therapy; FFS, fee-for-service; MAX, Medicaid analytic extract.

HIV status determined by two or more HIV diagnoses recorded on separate dates (International Classification of Diseases 9th Revision Code: 042, V08) or a minimum of two antiretroviral ingredients fill records. For persons with only one HIV diagnosis, persons were regarded as HIV positive if the diagnosis was from inpatient or long-term care records or if they had two or more CD4 count test records after HIV diagnosis or if had HIV-related dementia or wasting diagnosis codes. These two last diagnoses were included because of their high specificity.

[Appendix Figure 3] Construction of the analytic sample of HIV- and HIV+ persons with statin


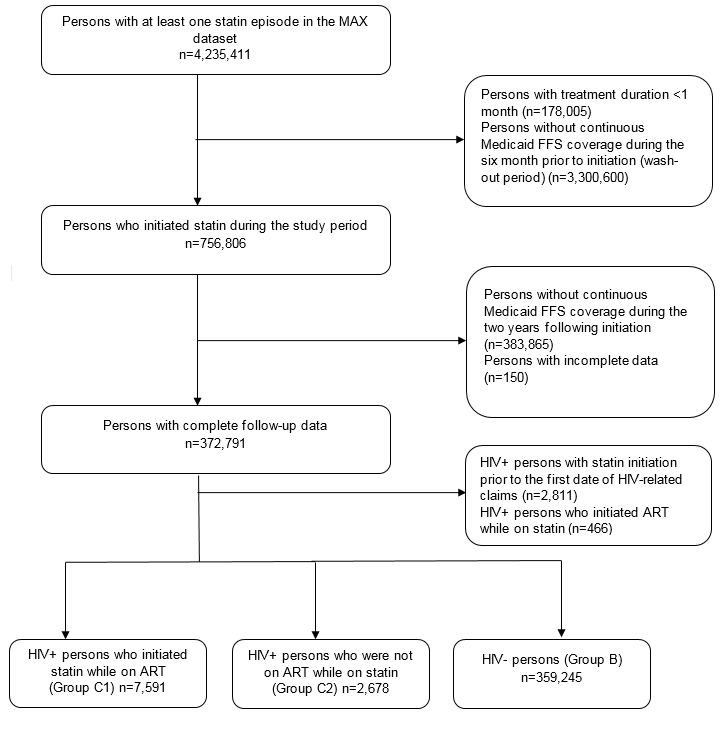


Abbreviations: ART, antiretroviral therapy; FFS, fee-for-service; MAX, Medicaid analytic extract.

[Appendix Figure 4] Construction of the analytic sample of HIV- and HIV+ persons with ACEI/ARB


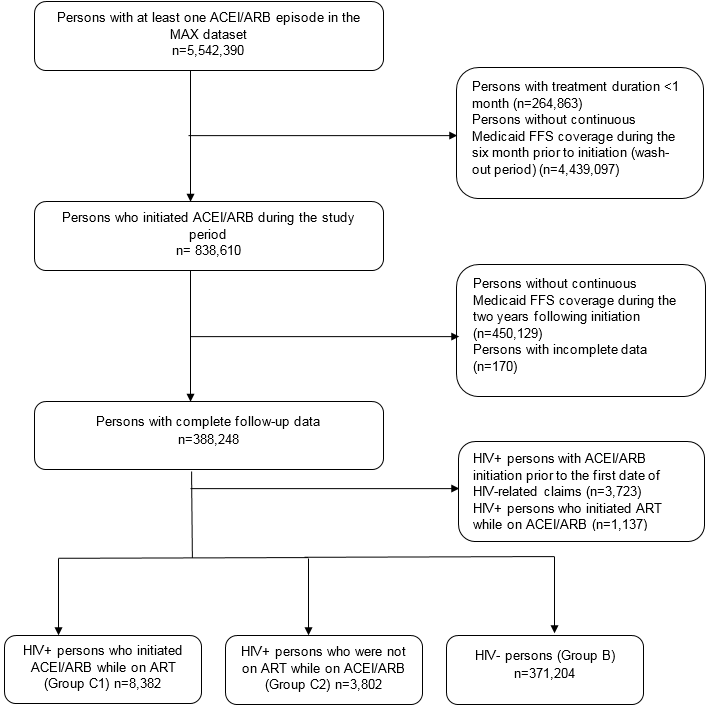


Abbreviations: ACEI, angiotensin-converting enzyme inhibitor; ARB, angiotensin receptor blocker; ART, antiretroviral therapy; FFS, fee-for-service; MAX, Medicaid analytic extract.

[Appendix Figure 5] Construction of the analytic sample of HIV- and HIV+ persons with metformin


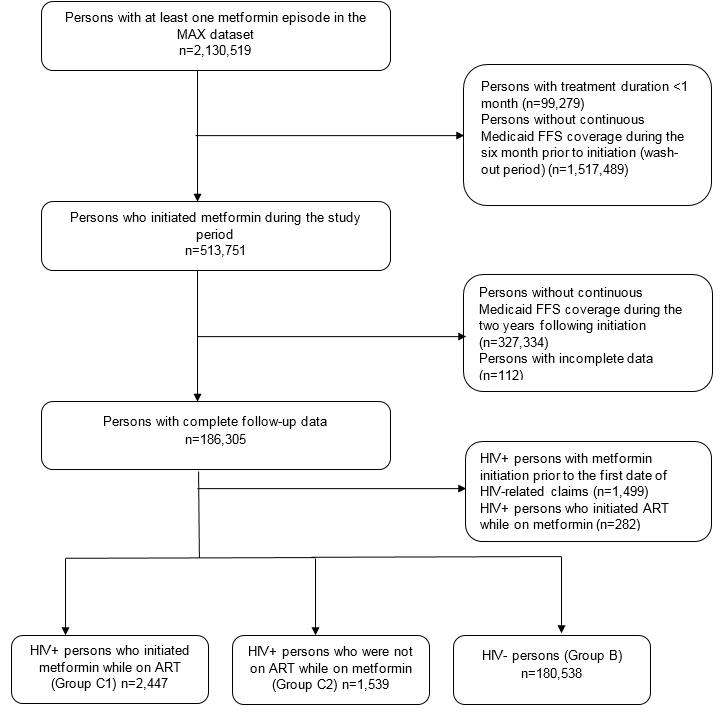


Abbreviations: ART, antiretroviral therapy; FFS, fee-for-service; MAX, Medicaid analytic extract.

[Appendix Table 1] Baseline characteristics of HIV+ persons who initiated statin, ACEI/ARB, or metformin

|  | **Statin** | | **ACEI/ARB** | | **Metformin** | |
| --- | --- | --- | --- | --- | --- | --- |
|  | **Group C1** | **Group C2** | **Group C1** | **Group C2** | **Group C1** | **Group C2** |
| **n** | 7,591 | 2,678 | 8,382 | 3,802 | 2,447 | 1,539 |
| **Treatment initiation year (%)** |  |  |  |  |  |  |
| 2001–2003 | 29.7 | 22.3 | 30.1 | 26.4 | 30.6 | 22.0 |
| 2004–2006 | 35.4 | 37.7 | 33.6 | 36.5 | 32.3 | 35.9 |
| 2007–2010 | 34.9 | 40.0 | 36.4 | 37.1 | 37.1 | 42.1 |
| **Age, years (%)** |  |  |  |  |  |  |
| <25 | 0.8 | 0.9 | 1.8 | 1.6 | 1.1 | 4.2 |
| 25–34 | 5.0 | 7.4 | 4.9 | 7.7 | 4.8 | 9.6 |
| 35–44 | 31.1 | 26.8 | 28.3 | 28.3 | 30.7 | 27.0 |
| 45–54 | 45.6 | 44.2 | 46.4 | 43.8 | 45.7 | 40.8 |
| 55+ | 17.6 | 20.7 | 18.6 | 18.6 | 17.7 | 18.4 |
| **Sex (% male)** | 57.5 | 47.1 | 58.1 | 45.8 | 50.4 | 41.5 |
| **Race/ethnicity (%)** |  |  |  |  |  |  |
| Asian/Pacific Islander/Native American | 1.4 | 1.9 | 1.3 | 1.8 | 1.8 | 1.6 |
| Black | 45.6 | 50.2 | 56.1 | 59.7 | 49.6 | 52.8 |
| Hispanic | 24.2 | 24.4 | 20.0 | 18.8 | 25.5 | 25.5 |
| Multiracial/Unknown | 6.4 | 5.6 | 6.2 | 5.8 | 5.7 | 5.7 |
| White | 22.4 | 18.0 | 16.4 | 14.0 | 17.4 | 14.4 |
| **State (%)** |  |  |  |  |  |  |
| California | 14.5 | 13.7 | 13.4 | 14.1 | 11.1 | 12.0 |
| Florida | 14.8 | 10.1 | 14.4 | 8.4 | 14.9 | 10.3 |
| Georgia | 4.5 | 3.4 | 4.9 | 5.2 | 4.1 | 4.1 |
| Illinois | 5.7 | 14.0 | 6.7 | 13.1 | 7.2 | 11.5 |
| Louisiana | 2.5 | 3.4 | 2.9 | 4.5 | 1.7 | 4.6 |
| Massachusetts | 4.1 | 2.8 | 3.9 | 2.7 | 5.5 | 3.3 |
| Maryland | 0.8 | 0.6 | 0.5 | 0.8 | 0.7 | 0.5 |
| North Carolina | 3.5 | 3.1 | 4.9 | 4.4 | 4.4 | 4.5 |
| New Jersey | 2.0 | 2.6 | 3.8 | 2.7 | 2.6 | 2.8 |
| New York | 44.4 | 42.4 | 42.0 | 39.9 | 44.7 | 43.1 |
| Ohio | 1.2 | 1.1 | 0.8 | 1.2 | 1.1 | 0.8 |
| Pennsylvania | 0.6 | 0.7 | 0.5 | 0.5 | 0.7 | 0.8 |
| Texas | 1.3 | 2.1 | 1.4 | 2.5 | 1.3 | 1.6 |
| Virginia | 0.2 | 0.2 | 0.1 | 14.1 | 0.1 | 0.3 |
| **Index regimen type (%)** |  |  |  |  |  |  |
| ACEI | - | - | 86.7 | 80.0 | - | - |
| ARB | - | - | 13.2 | 19.9 | - | - |
| ACEI + ARB | - | - | 0.2 | 0.1 | - | - |
| **Alcohol use (% yes)** | 34.5 | 52.1 | 41.2 | 54.9 | 41.4 | 56.0 |
| **Drug use (% yes)** | 45.3 | 61.8 | 54.5 | 65.9 | 51.7 | 65.6 |
| **Tobacco use (% yes)** | 34.8 | 48.1 | 38.7 | 46.5 | 38.8 | 48.4 |

Abbreviations: ACEI, angiotensin-converting enzyme inhibitor; ARB, angiotensin receptor blocker.

Group C1 includes HIV+ persons who initiated each medication while on antiretroviral therapy. Group C2 includes HIV+ persons who were not on ART while using these medications. Missing values in sex accounted for 0.03% of statin group C1, 0.06% of ACEI/ARB group C2, 0.08% of ACEI/ARB group C2, and 0.04% of metformin group C1.

[Appendix Table 2] Trends in antiretroviral therapy adherence using different outcome measurements

| **Treatment**  **initiation year** | **2001** | **2002** | **2003** | **2004** | **2005** | **2006** | **2007** | **2008** | **2009** | **2010** | **2011** | **2012** | **n** |
| --- | --- | --- | --- | --- | --- | --- | --- | --- | --- | --- | --- | --- | --- |
| >95% Implementation, two year^a^ | 22.7% | 27.4% | 29.0% | 32.6% | 31.8% | 34.5% | 37.5% | 36.3% | 36.8% | 36.2% | - | - | 23,343 |
| >80% Implementation, two year^a^ | 52.0% | 57.0% | 60.2% | 62.6% | 63.6% | 66.6% | 67.9% | 68.3% | 67.6% | 66.5% | - | - | 23,343 |
| Mean PDC, two years^a,b^ | 57.8% | 59.7% | 58.4% | 60.7% | 62.7% | 65.4% | 67.4% | 68.7% | 68.8% | 70.2% | - | - | 23,343 |
| >90% PDC, two years^a,b^ | 20.0% | 22.3% | 23.0% | 27.6% | 29.7% | 32.3% | 35.1% | 37.1% | 37.2% | 39.7% | - | - | 23,343 |
| >90% Implementation, one year^c^ | 33.9% | 39.6% | 42.7% | 45.5% | 46.0% | 48.6% | 51.1% | 51.0% | 52.3% | 52.5% | 51.4% | - | 33,754 |
| >90% Implementation, during persistent episode^d^ | 34.5% | 39.8% | 43.2% | 45.1% | 45.8% | 47.6% | 49.1% | 50.0% | 51.3% | 52.0% | 50.7% | 52.5% | 49,822 |
| Adjusted rate of >90% Implementation, two year^a,e^ | 34.8% | 39.1% | 42.8% | 45.2% | 45.8% | 46.1% | 48.9% | 48.4% | 50.3% | 54.4% | - | - | 23,343 |

Abbreviations: PDC, proportion of days covered.

^a^ During the two years following initiation, using the same cohort from the main analyses.

^b^ PDC rates are lower than implementation rates because non-persistent time is included in the denominator..

^c^ Percentage of persons with >90% implementation rate during the 1-year following initiation. Includes persons who had continuous Medicaid fee-for-service coverage during the 1-year following initiation.

^d^ Percentage of persons with >90% implementation rate during the persistent episode. Followed persons until the last prescription date, end of the study, death, or loss of Medicaid coverage, whichever came first, for the denominator of the rate.

^e^ Adjusted predicted rates using marginal standardization. Adjusted for all variables listed in main Table 3.

[Appendix Table 3] Adjusted odds of >90% proportion of days covered

|  | **OR** | **99% CI** | |
| --- | --- | --- | --- |
| **Treatment initiation year (ref=2001–2003)** |  |  |  |
| 2004–2006 | 1.11 | 1.00 | 1.25 |
| 2007–2010 | 1.25 | 1.08 | 1.45 |
| **Age, years (ref=55+)** |  |  |  |
| <25 | 0.59 | 0.49 | 0.71 |
| 25–34 | 0.48 | 0.41 | 0.57 |
| 35–44 | 0.69 | 0.60 | 0.80 |
| 45–54 | 0.85 | 0.74 | 0.98 |
| **Sex (ref=Female)** |  |  |  |
| Male | 1.15 | 1.06 | 1.24 |
| **Race/Ethnicity (ref=White)** |  |  |  |
| Black | 0.69 | 0.61 | 0.77 |
| Hispanic | 0.90 | 0.79 | 1.03 |
| Asian/Pacific Islander /Native American | 0.92 | 0.63 | 1.34 |
| Multi/unknown | 0.92 | 0.77 | 1.10 |
| **State (ref=New York)** |  |  |  |
| California | 1.14 | 1.01 | 1.29 |
| Florida | 0.72 | 0.62 | 0.83 |
| Georgia | 0.55 | 0.45 | 0.67 |
| Illinois | 0.90 | 0.77 | 1.05 |
| Louisiana | 0.45 | 0.36 | 0.57 |
| Massachusetts | 0.73 | 0.60 | 0.89 |
| Maryland | 0.66 | 0.40 | 1.09 |
| North Carolina | 0.85 | 0.71 | 1.02 |
| New Jersey | 1.08 | 0.86 | 1.36 |
| Ohio | 0.81 | 0.55 | 1.18 |
| Pennsylvania | 1.01 | 0.64 | 1.60 |
| Texas | 0.29 | 0.22 | 0.40 |
| Virginia | 0.61 | 0.28 | 1.30 |
| **NRTI backbone (ref=TDF/ABC)** |  |  |  |
| ZDV | 0.78 | 0.69 | 0.87 |
| DDI/D4T | 0.87 | 0.75 | 1.01 |
| Others | 0.95 | 0.67 | 1.34 |
| **Regimen type (ref=PI based)** |  |  |  |
| Boosted PI based | 1.16 | 1.01 | 1.33 |
| Integrase inhibitor based | 1.96 | 1.18 | 3.26 |
| NNRTI based | 1.06 | 0.91 | 1.23 |
| NRTI based | 0.61 | 0.49 | 0.76 |
| Multiple class | 1.13 | 0.89 | 1.43 |
| **ART pill burden (ref=10+)** |  |  |  |
| 1 | 2.35 | 1.76 | 3.12 |
| 2-3 | 1.99 | 1.63 | 2.43 |
| 4-5 | 1.46 | 1.22 | 1.74 |
| 6-9 | 1.25 | 1.07 | 1.46 |
| **Alcohol use (ref=no)** | 0.78 | 0.70 | 0.86 |
| **Drug use (ref=no)** | 0.75 | 0.67 | 0.83 |
| **Tobacco use (ref=no)** | 0.85 | 0.77 | 0.93 |

Abbreviations: ABC, abacavir; ART, antiretroviral therapy; CI, confidence interval; D4T, stavudine; DDI, didanosine; NNRTI, nonnucleoside reverse transcriptase inhibitor; NRTI, nucleoside reverse transcriptase inhibitors; OR, odds ratio; PI, protease inhibitor; Ref, reference category; TDF, tenofovir; ZDV, zidovudine.

Reference

1. Youn B, Shireman TI, Lee Y, Galarraga O, Rana AI, Justice AC, et al. Ten-year trends in antiretroviral therapy persistence among US Medicaid beneficiaries. Aids. 2017;31(12):1697-707.
